# Safety and Efficacy of Pulmonary Rehabilitation for Long COVID Patients Experiencing Long-Lasting Symptoms

**DOI:** 10.3390/ijerph21020242

**Published:** 2024-02-19

**Authors:** Espérance Moine, Virginie Molinier, Adriana Castanyer, Amandine Calvat, Guillaume Coste, Antonin Vernet, Audrey Faugé, Perrine Magrina, Joan Lluis Aliaga-Parera, Nicolas Oliver, François Alexandre, Nelly Heraud

**Affiliations:** 1 Direction de la Recherche Clinique et de l’Innovation en Santé, Clariane, 34700 Lodève, France; 2 Clinique du Souffle La Solane, Inicea, 66340 Osséja, France; 3 Clinique du Souffle La Vallonie, Inicea, 34700 Lodève, France

**Keywords:** long COVID, post-COVID-19 syndrome, multidisciplinary intervention, treatability, MCIDs

## Abstract

Due to the high prevalence and persistence of long COVID, it is important to evaluate the safety and efficacy of pulmonary rehabilitation (PR) for patients who experience long-lasting symptoms more than six months after initial COVID-19 onset. Enrolled patients were admitted for a four-week in-patient-PR due to long COVID symptoms (n = 47). The safety of PR was confirmed by the absence of adverse events. Symptom-related outcomes were evaluated pre- and post-PR with significant score changes for: 6 min walking distance (61 [28 to 103] m), quality of life (mental Short Form-12: 10 [6 to 13], and physical: 9 [6 to 12]), Montreal Cognitive Assessment (1 [0 to 3]), fatigue (MFI-20: −19 [−28 to −8]), dyspnea (DYSPNEA-12: −7 [−9 to −2] and mMRC; −1 [−1 to 0]), Nijmegen questionnaire (−8 [−11 to −5]), anxiety and depression (HADS:−4 [−5 to −2] and −2 [−4 to −1], respectively) and posttraumatic stress disorder checklist scale (−8 [−12 to −4]). At the individual level, the percentage of symptomatic patients for each outcome decreased, with a high response rate, and the number of persistent symptoms per patient was reduced from six at PR initiation to three at the end of the program. Our results show that in-PR is safe and efficient at decreasing long-lasting symptoms experienced by long COVID patients at more than six months after initial disease onset.

## 1. Introduction

Several months after COVID-19 infection, some patients suffer from persistent symptoms. This syndrome is called long COVID and was defined by the World Health Organization (WHO) in 2021 as “*the condition that occurs in individuals with a history of probable or confirmed SARS-CoV-2 infection, usually 3 months from the onset of COVID-19, with symptoms that last for at least 2 months and cannot be explained by an alternative diagnosis*” [1]. The prevalence of patients experiencing long COVID after being infected by SARS-CoV-2 is 30% [2,3,4], which amounts to more than two million people in France. A wide range of long COVID symptoms have been reported, and a single patient can have multiple symptoms [5,6]. Although the pathogenesis of long COVID remains unclear, several hypotheses have been discussed. Among them, the persistence of SARS-CoV-2 viral charges [7], reactivation of latent virus [8], immune system disorders [9], and inflammation leading to alterations of organs and tissues [10] are the most reported in the literature [11]. Although the reason why long COVID occurs in some and not others is not known, some risk factors have been identified, including female sex, belonging to an ethnic minority, socioeconomic deprivation, smoking, obesity, and having comorbidities [12]. Once long COVID has been triggered, complete recovery is unlikely as there is an 85% probability of symptom persistence at 12 months [13]. Among 53 persistent symptoms reported at one year of follow-up, the prevalence of fatigue, for example, remains very high for 81% of patients [13]. This persistence of long COVID symptoms can last well beyond a year, as also reported in other nearby respiratory infections: from 2 to 4 years after Severe Acute Respiratory Syndrome (SARS), Middle East Respiratory Syndrome (MERS) coronavirus infections, and the infectious episode for H7N9-A influenza virus [14,15,16,17].

In these previous epidemics, exercise training provided promising results in terms of reducing the impact of symptoms. For example, in recovering patients five weeks after SARS onset, a six-week exercise training program was effective at improving both the cardiorespiratory and musculoskeletal performances, whereas the control group did not exhibit significant improvement [18]. In order to reduce the persistent symptoms of long COVID, the WHO in 2021 encouraged countries to treat long COVID patients with pulmonary rehabilitation (PR) [19]. Pulmonary rehabilitation is a comprehensive multidisciplinary intervention recognized as the cornerstone of chronic respiratory disease management and mainly comprises exercise training and education. Among its main benefits, PR is efficient at reducing fatigue and dyspnea [20,21] and increasing patient quality of life [22], which are among the main repercussions of long COVID [23].

Several studies have reported a decrease in dyspnea and fatigue and improved exercise tolerance and quality of life after a PR program in patients who had been infected with SARS-CoV-2 [24,25,26]. Nevertheless, these studies all included patients close to the initial time of infection (1 to 6 months), and most of them did not include a control group. At such close proximity to the time of infection, this first implies the possibility of at least partial spontaneous recovery. Indeed, it has been shown that some post-COVID symptoms can progress favorably and spontaneously up to six months after infection [27] before reaching a plateau for patients whose symptoms persist [13]. Less than six months post-COVID, it is not possible to draw conclusions from such a study without a control group, as during this period, it is impossible to know whether the observed effect is due to the PR program and/or spontaneous recovery. Furthermore, the safety and relevance of PR and mostly exercise retraining are currently a matter of debate and, in opposition to the WHO recommendations, some authors warn of the focus of PR to treat long COVID patients, drawing a parallel with patients experiencing myalgic encephalomyelitis/chronic fatigue syndrome (ME/CFS) [11,28].

In light of all of these elements, it is important to evaluate the safety and relevance of a PR program for patients still experiencing long COVID symptoms several months after initial COVID-19 onset (>6 months). Thus, the aim of the present study was to assess the safety and evaluate the efficacy of a PR program for long COVID patients experiencing long-lasting symptoms (e.g., fatigue and/or dyspnea).

## 2. Materials and Methods

### 2.1. Participants

This prospective observational study was conducted at the Clinique du Souffle La Solane (Osséja, France) and the Clinique du Souffle La Vallonie (Lodève, France) pulmonary rehabilitation centers between February 2021 and March 2022. All patients admitted for inpatient PR treatment (in-PR) because of persistent or progressive respiratory symptoms or functional limitations after confirmed COVID-19 infection (long COVID symptoms) were assessed for eligibility. The inclusion criteria were patients older than 18 years who had documented COVID-19 infection (polymerase chain reaction test (PCR), antigen test, or serology analysis), experiencing persistent symptoms for at least six months after the initial COVID-19 infection and with a confirmed association between persistent symptoms and COVID infection using clinical examination with a medical doctor at PR entry by studying the history of the disease and the patient’s history in order to rule out any other potential cause for the presence of these symptoms. The exclusion criteria were patients without a confirmed long COVID diagnosis, patients following an outpatient PR program, patients with a delay between acute COVID-19 infection and PR entry lower than six months, and patients who had expressed opposition to the use of their data.

In accordance with the French legislation for prospective studies collecting only routine care data (CNIL, reference methodology n°004, Deliberation n° 2018-155 of 3 May 2018), selected patients were informed of the use of their data for research purposes and could choose not to be involved in the study. The protocol was deposited in the French Hub Data Health platform before the beginning of the analyses (F20210604170633).

### 2.2. Pulmonary Rehabilitation Program

Patients underwent multidisciplinary and individualized inpatient rehabilitation according to the statements for pulmonary rehabilitation programs [29]. The four-week PR program harmonized between the two clinics and met common specifications, including a mean of 26 individualized endurance training sessions combining walking and ergocycling at an intensity close to the ventilation threshold and 12 individualized resistance training sessions. The ATR/ERS recommendations are to exercise at intensities between 8 and 12 repetitions [29]. Therefore, patients were asked to work at a level of perceived exertion of 7 on 10, thanks to the correspondence established between perceived effort intensity and actual effort intensity [30]. Each resistance training session included four exercises: two exercises targeting the muscles of the lower limbs and two exercises targeting the muscles of the upper limbs. A fundamental aspect of the program support consisted of individualized patient education, dyspnea management, and psychosocial counseling (a mean of eight sessions).

### 2.3. Procedures and Data Collection

#### 2.3.1. Clinical Data

Data on patient demographics such as age, sex, body mass index, number and type of comorbidities, and the PR starting date were obtained from the patient’s medical records, as was COVID-related information such as the date and the severity of the initial COVID-19 infection.

Clinical respiratory markers were measured upon initiation of the PR (T1): the forced expiratory volume in one second (FEV_1_), forced vital capacity (FVC), total lung capacity (TLC), and blood gases (PaO_2_ and PaCO_2_). Calculation of the predicted FEV_1_ and predicted TLC were performed automatically during the plethysmography, in accordance with The Global Lung Initiative of the European Respiratory Society recommendations [31,32,33].

As recommended, several validated questionnaires and tests relating to the main symptoms of long COVID were carried out upon initiation (T1) and termination (T2) of the PR [34]: the experienced long COVID symptoms and the most disabling symptom, dyspnea physical-affective items (DYSPNEA-12 questionnaire), and dyspnea impact (modified Medical Research Council Dyspnea Scale (mMRC)), fatigue (Multidimensional Fatigue Inventory (MFI-20) questionnaire), anxiety and depression (Hospital Anxiety and Depression Scale (HADS) questionnaire), posttraumatic stress (Posttraumatic Stress Disorder Checklist Scale (PCLS) questionnaire), hyperventilation (Nijmegen questionnaire, NQ), cognitive functions (Montreal Cognitive Assessment (MoCA) questionnaire), health-related quality of life (SF-12 questionnaire), exercise tolerance (6 min Walking Distance (6MWD) test), and muscle function (quadriceps isometric maximal strength (QMVC)). Details regarding references [29,35,36,37,38,39,40,41,42,43,44,45,46,47,48], administration, and scoring for each test and questionnaire are provided in Supplementary Data S1.

#### 2.3.2. Safety Data

The safety of PR for long COVID patients was evaluated using the number of adverse events (increased fatigue, muscle injury, deteriorated health status compared to the entry) quantified during the program, as well as the percentage of patients who deteriorated on each outcome. The Minimal Clinically Important Difference (MCID) is defined as the smallest change in score that patients perceive as beneficial or detrimental, and it is useful in the clinical interpretation of health status data, particularly in response to an intervention. Deterioration was assessed using the worsening MCID, when available for each indicator in the literature (Table 1), and was defined by a deterioration in the individual response on a given indicator exceeding the available worsening MCID between T1 and T2. In the absence of specific worsening MCID, the reported Minimal Detectable Change (MDC) was used to qualify the deteriorated patients. The MDC is defined as the smallest change for an individual that is likely to be beyond the measurement error of the scoring tool and represent true statistical change. This indicator is adapted for individual analysis and can be used to determine deterioration [49]. By contrast, classical MCIDs, which are developed to perceive a beneficial change only, can be smaller or larger when assessing a deterioration and are, therefore, not suitable for that purpose [50,51,52,53,54].

#### 2.3.3. Efficacy Data

The evaluation of the efficacy of PR for long COVID patients was performed on the complete cohort and individually. The individual analyses were performed only regarding treatable symptoms for each patient. To assess the treatability of a symptom, cut-off scores for each outcome were used when available in the literature (Table 2) [63]. For each treatable symptom, we assessed the percentage of responders and the percentage of patients in remission at the end of the PR. A patient was considered as responder on one outcome when the change in their score between the start and end of the PR (T1 versus T2) was higher than the available MCIDs designed to assess an improvement (Table 2). A patient was considered in remission on one outcome (i.e., no longer symptomatic) when their score at the end of the PR (T2) exceeded the cut-off score.

### 2.4. Statistical Analyses

All statistical analyses were performed using Statistica software (StatSoft, Inc., version 6.0, Tulsa, OK, USA).

The data are reported as means and the standard deviation (SD) in case of normal distribution (test with Q-Qplot, Shapiro–Wilk, Skewness, Kurtosis), or as *medians [lower quartile (LQ) to upper quartile (UQ)]* otherwise. Changes in outcome scores are represented as means [lower confidence interval at 95% (LCI95) to upper confidence interval at 95% (UCI95)] in case of normal distribution or as *medians [LQ to UQ]* otherwise.

Paired sample t-tests were performed to examine the effects of PR on the various outcomes. Student’s *t*-tests were used when the variables exhibited a normal distribution, or Wilcoxon rank tests otherwise. To investigate the associations between change in outcomes after PR and the delay between the acute COVID-19 infection and PR initiation, their correlations were assessed with Pearson’s test or Spearman’s test according to the normality of the distributions.

## 3. Results

### 3.1. Population Characteristics at Baseline

**Figure 1:**
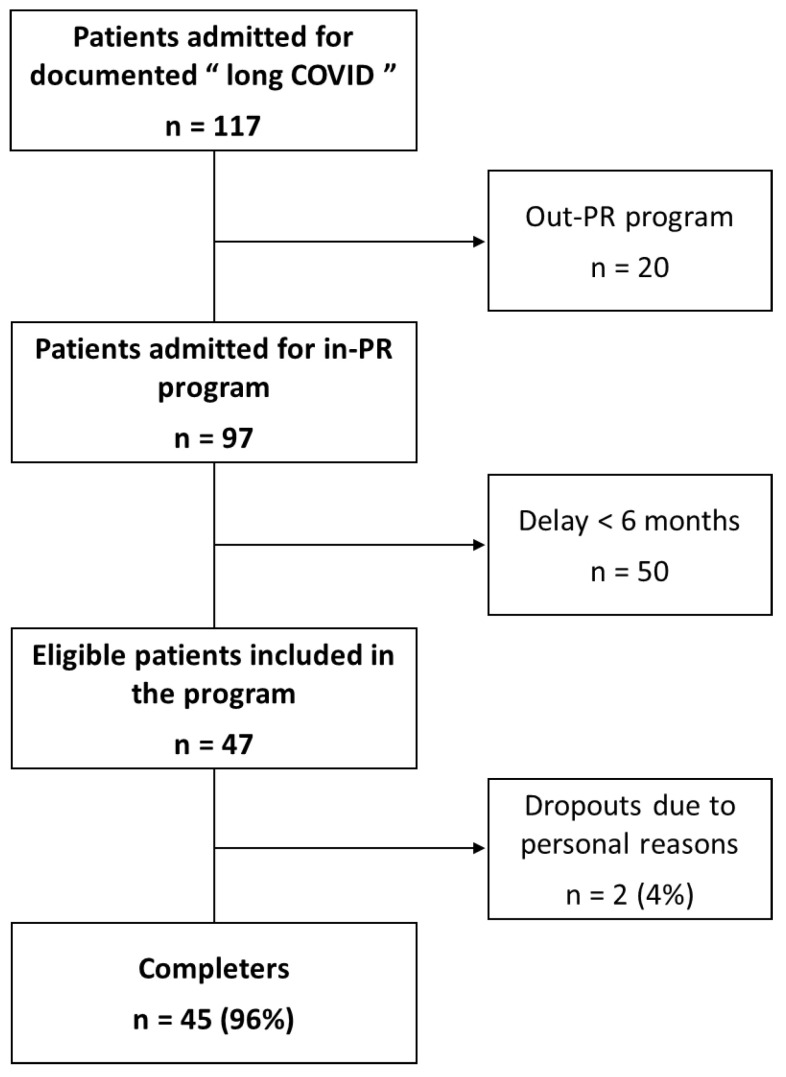
Flowchart of the study.

As detailed in Figure 1, a total of 117 consecutive patients were admitted for “long COVID” during the inclusion period. Of these, 20 patients were not included in the study as they followed an out-PR program, and 50 patients had a delay of less than six months between acute disease onset and PR initiation.

Forty-seven patients admitted for in-PR due to long COVID symptoms were enrolled: 8 patients participated in the PR program at the Clinique du Souffle La Vallonie and 39 at the Clinique du Souffle La Solane. Two patients did not complete the program due to personal reasons. The descriptive characteristics of the participants are reported in Table 3. Reported respiratory comorbidities were sleep apnea (n = 8), asthma (n = 6), chronic obstructive pulmonary disease (n = 5), and emphysema (n = 2). Cardiovascular comorbidities were high blood pressure (n = 6), heart disease (n = 6), and pericarditis (n = 1). Metabolic-associated comorbidities were obesity (n = 12) and diabetes (n = 5).

### 3.2. Safety of a PR Program for Long COVID Patients

No adverse events were reported for the 96% of patients who completed the program. Symptom deterioration was found for the physical dimension of quality of life (n = 1; scores at T1 = 66 and T2 = 53) and physical-affective dyspnea (n = 2; scores at T1 = 0 and T2 = 4 for both). The three cases of deterioration were found in three different patients, and thus, none of the patients experienced deterioration on more than one outcome at the same time. No deterioration was reported for exercise tolerance, muscle strength, the mental dimension of quality of life, fatigue, dyspnea impact, anxiety, depression, or cognition. 

### 3.3. Efficacy of the PR Program on the Study Outcomes

The overall effects of the PR on the study outcomes are detailed in Table 4. Exercise tolerance, health-related quality of life, fatigue, dyspnea (impact, physical and affective dimensions), hyperventilation, anxiety, depression, posttraumatic stress disorder, and cognition significantly improved at the end of the PR (*p*-values < 0.001 and *p*-value of MoCA and mMRC < 0.01). The reported changes were not statistically significant for muscle strength (*p*-value = 0.10).

**Figure 2:**
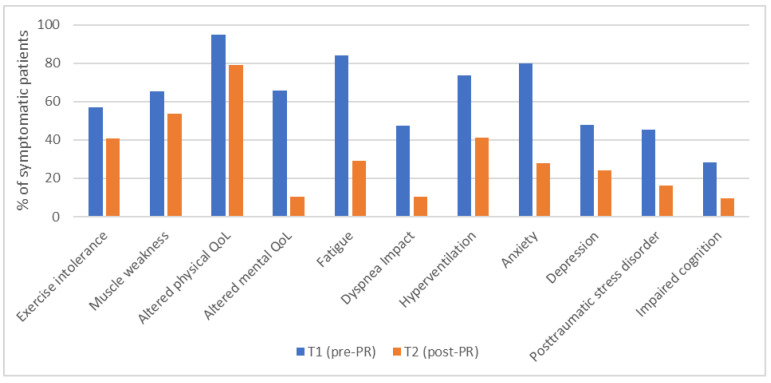
Histogram representing the percentage of symptomatic patients at T1 (pre-PR) and T2 (post-PR). PR: Pulmonary Rehabilitation. Exercise intolerance (n = 44); Muscle weakness (n = 26); Altered physical QoL (n = 38); Altered mental QoL (n = 38); Fatigue (n = 38); Dyspnea impact (n = 19); Hyperventilation (n = 34); Anxiety (n = 25); Depression (n = 25); Posttraumatic stress disorder (n = 31); Impaired cognition (n = 32).

Regarding the individual responses, the percentage of symptomatic patients decreased for all symptoms. As shown in Figure 2, there was a decrease in symptomatic patients at the end of the PR for exercise intolerance (−28%), muscle weakness (−18%), altered physical (−17%) and mental (−84%) health-related quality of life, fatigue (−66%), dyspnea impact (−78%), hyperventilation (−44%), anxiety (−65%), depression (−50%), posttraumatic stress disorder (−64%), and impaired cognition (−67%). Overall, the average number of assessed symptoms per patient fell from 5.5 ± 2.3 at PR initiation to 2.7 ± 1.9 at the end of the PR. 

**Figure 3:**
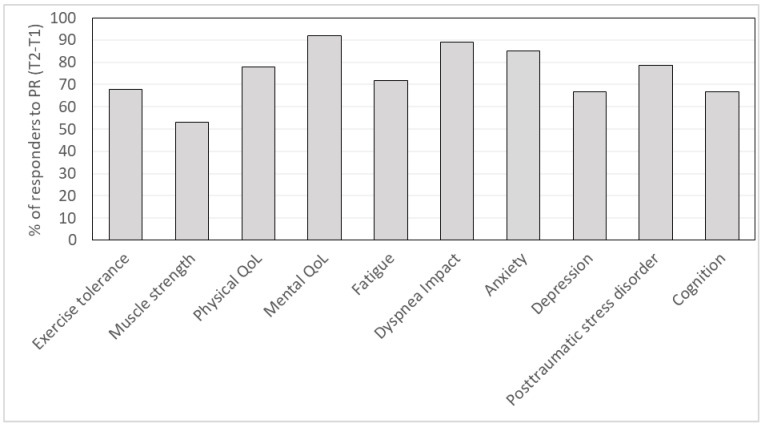
Histogram of the percentage of responders for the evaluated outcomes among the treatable patients. PR: Pulmonary Rehabilitation. Exercise tolerance (n = 25); Muscle strength (n = 17); Physical QoL (quality of life) (n = 36); Mental QoL (quality of life) (n = 25); Fatigue (n = 32); Dyspnea impact (n = 9); Anxiety (n = 20); Depression (n = 12); Posttraumatic stress disorder (n = 14); Cognition (n = 9).

As shown in Figure 3, the majority of patients responded to PR in terms of their long COVID symptoms, ranging from 53% up to 92% according to the evaluated symptom.

The correlations between the delay in the first symptoms of acute COVID and PR enrolment and change in outcomes were assessed and are presented in Table 5. There were no significant correlations (p-values > 0.05) for any of the indicators.

## 4. Discussion

This study highlights the efficacy of PR in relieving the main long COVID symptoms. Indeed, the PR program was able to significantly reduce the long-lasting symptoms and increase health-related quality of life and exercise tolerance in long COVID patients. Moreover, the number of measured symptoms that patients experienced decreased, from six at PR initiation to a mean of three symptoms per patient at the end of the PR out of an average of eight symptoms evaluated. Furthermore, PR appears to be safe for long COVID patients as no adverse events were reported among the 96% of patients who completed the program. It is also interesting to note that only 3 cases among the 47 patients and the 12 evaluated outcomes were marked by partial clinical deterioration. The first patient showed a deterioration in the physical dimension of health-related quality of life (SF-12 PCS), but the scores at T1 (PR entry) and T2 (end of PR) are both higher than the cut-off score (i.e., not symptomatic if score > 50 units) meaning no health-related quality of life issues for this patient. The two other patients showed deterioration on the multidimensional dyspnea assessment (evaluated via the DYSPNEA-12 questionnaire and scored on 36 units). Their score was equal to 0 at T1, meaning no issues with this symptom. At T2, their scores reached 4/36, which is, nevertheless, low on the scale of DYSPNEA-12. However, as no cut-off score is available to categorize symptomatic patients on this scale, we cannot affirm that these patients are still non-symptomatic at T2. Unfortunately, the first patient did not perform the alternative dyspnea impact evaluation (mMRC), but the second patient performed the mMRC evaluation and was non-symptomatic on the dyspnea impact scale (neither in T1 nor in T2), allowing us to believe that the slight deterioration observed on the DYSPNEA-12 scale is not representative of a real deterioration in the state of health of this patient. Moreover, if the dyspnea of these two patients had worsened symptomatically, it should have been accompanied by other repercussions (such as worsening quality of life or anxiety), which is not the case here. To conclude, in order to be fully transparent on the safety results of this study, we chose to report these three slight deteriorations. However, looking at the individual scoring of these three patients highlights that there are minor deteriorations on non-symptomatic patients with no issues.

Participant characteristics in our study are consistent with the reported risk factors associated with the development of long COVID (i.e., women, obesity, comorbidities, etc.) [79]. It should also be noted that the included patients reported a wide range of symptoms, with a total of 24 different self-reported symptoms. This broad symptomology is in accordance with the variety of symptoms reported in the literature [23,79,80]. However, our study highlighted two exceptions: one for post-exertional malaise and the other for respiratory symptoms. Indeed, only one patient among the 47 included in the study reported post-exertional malaise, which contrasts with a recent study that reported up to 73% prevalence at six months post-infection [81]. The low occurrence of this symptom in our population may be partly explained using two factors: firstly, this symptom was not clearly listed in the symptom list offered to patients even if there was an “other” box offering the possibility to add non-listed symptoms; and secondly, it is likely that a lack of familiarity with PEM lead several patients to assimilate this to fatigue and therefore was not able to distinguish both symptoms. This issue has been described, and a specific questionnaire, such as the DePaul Symptom Questionnaire-Post-Exertional Malaise, should be used to assess the prevalence of this symptom [82]. Concerning the relatively high prevalence of respiratory symptoms [23,82,83], this may be linked to an orientation bias of patients by prescribers. Indeed, the program was carried out in a center recognized nationally for its expertise in the management of chronic respiratory diseases, and thus, it is likely that long COVID patients with a high prevalence of respiratory disorders were preferentially referred to this center.

Despite the growing number of studies on the benefits of PR for post-COVID patients, to the best of our knowledge, this is the first study to have assessed the safety of a PR program for long COVID patients. Yet, safety issues have been raised in the literature, particularly in the context of PR programs, pointing out the urgency of assessing this [11,28]. Interestingly, no adverse events were reported during the study for the 96% of individuals who completed the program. Moreover, the analyses highlighted only three cases of deterioration in the 47 patients and the 12 evaluated outcomes. It is also interesting to note that the three patients deteriorated on only one outcome, with all exhibiting improvement for other parameters. Accordingly, although we cannot overlook the fact that the scores for some parameters were degraded at the end of the PR for these patients, the conclusion that PR has deteriorated their health would be clearly exaggerated. As there were few outcomes that were likely to deteriorate (only three cases of deterioration in total), we can conclude that pulmonary rehabilitation appears to be a safe intervention for patients experiencing long COVID symptoms.

To examine the efficacy of PR, we first carried out global pre/post analyses in order to assess the changes occurring during the program on the various outcomes measured. However, as a mean analysis can obscure the inter-individual differences, analysis of the individual responses of the patients for each outcome was, therefore, necessary. In this study, individual responses were assessed only for patients who were symptomatic for the outcome of interest upon initiation of the PR. Indeed, any given patient was not necessarily symptomatic for all the analyzed outcomes, and individual responses to PR for a non-symptomatic outcome were not clinically relevant to assess PR efficacy [58].

Regarding the overall pre/post analyses, the results highlight significant improvement in the patients for all measured outcomes at the end of the PR, except for muscle strength. However, at the individual level, upon initiation of the program, the majority of the symptomatic patients had significantly increased muscle strength, as 53% exhibited an increase greater than the MCID. This prevalence of patient responses regarding muscle strength (i.e., 53%) is consistent with the study by Desachy et al., who reported a 50% response rate to QMVC at the end of a PR program in patients with chronic obstructive pulmonary disease [83]. The heterogeneity of responses to muscle strength is an important issue of current PR programs, as seen here for long COVID patients, and it highlights the importance of analyzing individual responses to conclude regarding PR (in)effectiveness and to consider care tailored to each patient. Aside from muscle strength, our results revealed an average degree of improvement for all of the other outcomes.

The individual analyses were consistent with the mean results pointing to a high rate of responders and a reduction in the number of symptomatic patients for all the outcomes, with an average of three persistent symptoms at the end of the program compared to six at the time of initiation of the PR. Taken together, these results confirm the efficacy of a PR program of only four weeks, with notably two-thirds of the patients being in remission for fatigue, which is the most reported symptom of long COVID [13,23,27], and 78% of the patients being in remission in terms of the dyspnea impact. Moreover, the program relieved two-thirds of the anxious patients, and more than 80% of patients with a deteriorated health-related quality of life before the PR had a normal quality of life after the PR.

Nevertheless, to confirm that the observed effects were indeed due to the program, and in the absence of a control group, correlation analyses between changes in long COVID symptoms and the delay between the first symptoms and the PR were performed. The results of this analysis did not reveal any significant correlations for the twelve outcomes that were assessed. This result reinforces the fact that the reported progressions of the patients are indeed due to the program benefits and not to spontaneous recovery over time.

Thus, our results demonstrate that PR, in this multidisciplinary approach, is an effective intervention for treating the main symptoms of long-term COVID in patients who nevertheless exhibited stabilized symptoms for more than six months [27]. Yet, despite the high percentage of responders recorded in this study, a total response or full remission of all the patients on the assessed outcomes was not achieved. However, the presence of non-responding patients is something commonly reported in PR and ranges between 30% to 50% of patients, depending on the outcomes and study [84,85]. In addition, patients can be responders on one or several measured outcomes but are not necessarily responders to all PR outcomes [86]. Therefore, it would be interesting to carry out additional work in order to identify the determinants of non-responding patients and accordingly consider alternatives to strengthen the efficacy of PR for all patients. Further studies should also consider potential delayed positive effects on patient health. Follow-up after PR could be relevant to assess such delayed positive effects, as well as how long the benefits of such a program persist. 

### Methodological Considerations

The first limitation of this study is the absence of a cut-off score available in the literature to categorize the symptomatic/asymptomatic patients via the DYSPNEA-12 questionnaire, thereby preventing us from performing individual analyses on this outcome. A second limitation of this study is the reliance on the MCIDs to discriminate responders from non-responders. As no MCIDs specific to long COVID patients were available, we used MCIDs published for other diseases, which may not be disease-adjusted. Indeed, using MCIDs available for other diseases may overestimate or underestimate the number of responders for an outcome or a symptom but provides an approximation of the number of patients for whom the program produces results. Moreover, as worsening MCIDs are scarce in the literature, MDCs were used here to assess patients likely to deteriorate at the end of PR. Nevertheless, using these indicators to assess the presence of symptoms as well as individual responses to the PR program was of the greatest interest from a clinical point of view. A third limitation of this study lies in the interpretation of safety data regarding the low prevalence of patients with post-exercise malaise. Indeed, we cannot generalize for this population based on a single case. A fourth limitation of this study is that the 6-minute walking test was performed only once, at the time of initiation of the PR, for 83% of the patients. Therefore, there is a risk that a learning effect influenced the 6MWD score at the end of the PR, which could lead to an overestimation of the program’s efficacy on exercise tolerance [87]. However, in our sample, 65% of the patients exceeded the MCID + learning effect threshold, thus supporting a genuine improvement in exercise tolerance for most of them. 

## 5. Conclusions

In patients experiencing long COVID symptoms (>6 months after acute disease onset), our study showed that a four-week in-PR program was safe, with only three cases of partial deterioration (out of 47 patients and 12 evaluated outcomes) and no adverse events. Moreover, the PR improved the main symptoms of long COVID (i.e., fatigue, dyspnea, exercise intolerance, quality of life, anxiety, etc.), with the majority of symptomatic patients being responders in terms of these symptoms at the end of the program. A reduction in the number of long COVID-associated symptoms was observed, ranging from six at the time of PR initiation and decreasing to an average of three per patient at the end of the PR. Overall, in light of its safety and efficacy, in-PR appears to be a genuine therapeutic option and has the potential to become a first-line treatment for patients suffering from long COVID with stabilized symptoms.

## Figures and Tables

**Table 1:** Worsening Minimal Clinically Important Differences (MCIDs) and Minimal Detectable Changes (MDCs) for the assessment of deterioration after PR, defined in the literature for the evaluated symptoms of this study.

Indicators	Worsening MCIDs/MDCs (Deteriorated)
Exercise tolerance (6MWD)	−30 m * [55]
Muscle strength (QMVC)	−25.02 Nm [56]
Quality of life (SF-12) physical	−8.9 units [49]
Quality of life (SF-12) mental	−13.8 units [49]
Fatigue (MFI-20) Total score	+15 units [57]
Dyspnea physical-affective (D-12)	+2.3 units [58]
Dyspnea impact (mMRC)	+1.2 units [59]
Hyperventilation (NQ)	NA
Anxiety (HADS-A)	+3.8 units [60,61]
Depression (HADS-D)	+3.99 units [60,61]
Posttraumatic stress disorder (PCLS)	NA
Cognition (MoCA)	4.21 units [62]

PR: Pulmonary Rehabilitation, NA: not available, 6MWD: 6 min Walking Distance, QMVC: Quadriceps Isometric Maximal Strength, SF-12: health-related quality of life, MFI: Multidimensional Fatigue Inventory, D-12: Physical-affective dyspnea questionnaire, mMRC: modified Medical Research Council Dyspnea Scale, NQ: Nijmegen questionnaire, HADS: Hospital Anxiety and Depression Scale, PCLS: Posttraumatic Stress Disorder Checklist Scale, MoCA: Montreal Cognitive Assessment.* Corresponds to worsening MCID; otherwise, MDCs for deterioration were used.

**Table 2:** Cut-off scores for the assessment of treatability/presence of symptoms and Minimal Clinically Important Differences (MCIDs) for responsiveness to PR, defined in the literature for the symptoms evaluated in this study.

Indicators	Cut-Off Scores	MCIDs (Responders)
Exercise tolerance (6MWD)	Intolerance if <82% of theoretical [64]	+ 35 m [65]
Muscle Strength (QMVC)	Weakness if <80% of theoretical [66]	+ 7.5 Nm [67]
QoL (SF-12) physical	Altered if <50 units [47]	+ 3 units [68]
QoL (SF-12) mental	Altered if <42 units [47]	+ 3.5 units [68]
Fatigue (MFI-20) Total score	>55 units [69]	−14.3 units [70,71]
Dyspnea physical-affective (D-12)	NA	−3 units [58]
Dyspnea impact (mMRC)	≥2 units [72]	−1 unit [73]
Hyperventilation (NQ)	>23 units [45]	NA
Anxiety (HADS-A)	≥8 units [74]	−2 units [75]
Depression (HADS-D)	≥8 units [74]	−1.8 units [75]
Posttraumatic stress disorder (PCLS)	≥44 units [76]	−7.9 units [77]
Cognition (MoCA)	<26 units [46]	+2 units [78]

PR: Pulmonary Rehabilitation, NA: not available, 6MWD: 6 min Walking Distance, QMVC: Quadriceps Isometric Maximal Strength, SF-12: health-related quality of life, QoL: Quality of Life, MFI: Multidimensional Fatigue Inventory, D-12: Physical-affective dyspnea questionnaire, mMRC: modified Medical Research Council Dyspnea Scale, NQ: Nijmegen questionnaire, HADS: Hospital Anxiety and Depression Scale, PCLS: Posttraumatic Stress Disorder Checklist Scale, MoCA: Montreal Cognitive Assessment.

**Table 3:** Descriptive characteristics of the sample.

	Mean (Standard Deviation)/*Median [LQ to UQ]*
**Baseline characteristics**	
Patients	n = 47
Age (years)	51 (12.6)
Female sex, n (%)	29 (62)
BMI (kg.m^−2^)	29.43 (6.88)
Number of comorbidities per patient	None (n = 13); 1 (n = 12); 2 (n = 8); 3 (n = 7); 4 (n = 5); 5 (n = 1); 6 (n = 1)
Main type of comorbidities	Respiratory (n = 18); cardiovascular (n = 14); metabolic (n = 13)
**Respiratory parameters at T1 (pre-PR)**	
FEV_1_ (L)	2.84 (0.78)
FEV_1_ (% predicted)	98.50 (23.34)
PaO_2_ (mmHg); n = 33	75.69 (17.62)
PaCO_2_ (mmHg); n = 33	36.84 (4.23)
FEV_1_/FVC	0.80 (0.12)
TLC (L); n = 46	6.24 (1.44)
TLC (% predicted); n = 45	112.27 (20.37)
**Acute COVID-19 characteristics**	
Diagnostic	PCR (n = 34); serology (n = 5); clinical diagnosis (n = 5); chest scan (n = 2); X-ray (n = 1)
Initial severity of acute COVID	Home care (n = 33), Hospitalization (n = 12); ICU (n = 2)
Delay between first symptoms and PR	*13 months [7 to 16]*
**Self-reported long COVID symptoms**	
Related to lung disease *	Dyspnea (n = 43); Chest pains (n = 20); Cough (n = 14); Sore throat (n = 5)
Cardiovascular *	Palpitations (n = 10)
Neurological *	Difficulty in concentrating and remembering (n = 25); Headache (n = 20); Sleep disorder (n = 14); Anxiety (n = 14); Irritability (n = 7); Loss of appetite (n = 5); Anosmia (n = 1); Depression (n = 1)
Physical *	Fatigue (n = 36); Feeling of muscle weakness (n = 21); Musculoskeletal pain (n = 19); Paraesthesia (n = 10); Itching (n = 8); Burning sensation (n = 7); Diarrhoea and vomiting (n = 4); Abdominal pain (n = 3); Regular chills (n = 3); Dysphonia (n = 4); Dizziness (n = 3); Eyesight problems (n = 2)
Most disabling symptom	Dyspnea (n = 21); Fatigue (n = 16); Musculoskeletal pain (n = 4); Chest pain (n = 2); Anxiety (n = 1); Post-exercise malaise (n = 1); Difficulty concentrating and remembering (n = 1); none (n = 1).

Note: total n = 47, BMI: Body mass index, PR: Pulmonary rehabilitation, FEV_1_: Forced expiratory volume in one second, PaO_2_: Partial pressure of arterial oxygen, PaCO_2_: Arterial partial pressure of carbon dioxide, FVC: Forced vital capacity, TLC: Total lung capacity, PCR: Polymerase chain reaction, ICU: Intensive care unit. * The same patient can have several symptoms.

**Table 4:** Effect of PR on study outcomes.

	T1 (Pre-PR)	T2 (Post-PR)	Change	*p*-Value
**Exercise performance**				
Exercise tolerance (6MWD—m) n = 44	519 (116)	589 (124)	*61 [28 to 103]*	***p* < 0.001**
Muscle strength (QMVC—Nm) n = 26	90 (36)	96 (31)	6 [−1 to 12]	*p* = 0.10 NS
Muscle strength (% predicted) n = 26	72 (23)	77 (22)	5 [−1 to 11]	*p* = 0.07 NS
**Health-related quality of life (SF-12)**				
Mental dimensions n = 38	40 (10)	50 (8)	10 [6 to 13]	***p* < 0.001**
Physical dimensions n = 38	33 (11)	42 (9)	9 [6 to 12]	***p* < 0.001**
**Respiratory and physical outcomes**				
Fatigue (MFI-20) n = 38	*70 [64 to 77]*	*48 [39 to 57]*	*−19 [−28 to −8]*	***p* < 0.001**
Dyspnea physical-affective (D-12) n = 38	*17 [12 to 20]*	*9 [4 to 12]*	*−7 [−9* to *−2]*	***p* < 0.001**
Dyspnea impact (mMRC) n = 25	*1 [1 to 2]*	*1 [1 to 1]*	*−1 [−1 to 0]*	***p* < 0.01**
Hyperventilation (Nijmegen) n = 34	28 (12)	20 (10)	−8 [−11 to −5]	***p* < 0.001**
**Psychological and cognitive outcomes**				
Anxiety (HADS-A) n = 25	10 (4)	6 (4)	−4 [−5 to −2]	***p* < 0.001**
Depression (HADS-D) n = 25	8 (4)	6 (4)	−2 [−4 to −1]	***p* < 0.001**
Posttraumatic stress disorder (PCLS) n = 31	41 (12)	33 (11)	−8 [−12 to −4]	***p* < 0.001**
Cognition (MoCA) n = 32	*27 [25 to 28]*	*28 [27 to 29]*	*1 [0 to 3]*	***p* < 0.01**

The data at T1 and T2 are presented as means (SD) or *medians [LQ to UQ]*. Changes are presented as means [LCI95 to UCI95] or *medians [LQ to UQ]*. NS = not significant, PR: Pulmonary Rehabilitation, 6MWD: 6 min Walking Distance, QMVC: Quadriceps Isometric Maximal Strength, SF-12: Health-related quality of life, MFI: Multidimensional Fatigue Inventory, D-12: DYSPNEA-12 questionnaire, mMRC: modified Medical Research Council Dyspnea Scale, HADS: Hospital Anxiety and Depression Scale, PCLS: Posttraumatic Stress Disorder Checklist Scale, MoCA: Montreal Cognitive Assessment.

**Table 5:** Correlations between change in outcomes and the delay between first symptoms and PR.

Outcomes	Delay between 1st Symptoms and PR Initiation
Exercise tolerance (6MWD)	−0.19 ns
Muscle strength (QMVC)	−0.11 ns
Physical quality of life (SF-12 PCS)	0.03 ns
Mental quality of life (SF-12 MCS)	0.12 ns
Fatigue (MFI-20)	0.27 ns
Dyspnea sensory-affective (D-12)	0.07 ns
Dyspnea impact (mMRC)	−0.04 ns
Hyperventilation (NQ)	0.02 ns
Anxiety (HADS-A)	−0.11 ns
Depression (HADS-D)	0.20 ns
Posttraumatic stress disorder (PCLS)	0.09 ns
Cognition (MoCA)	−0.08 ns

The outcome scores are expressed as the difference between T2 and T1. ns = not significant. PR: Pulmonary Rehabilitation, 6MWD: 6 min Walking Distance, QMVC: Quadriceps Isometric Maximal Strength, SF-12: Health-related quality of life, MFI: Multidimensional Fatigue Inventory, D-12: DYSPNEA-12 questionnaire, mMRC: modified Medical Research Council dyspnea scale, NQ: Nijmegen questionnaire, HADS: Hospital Anxiety and Depression Scale, PCLS: Posttraumatic stress disorder Checklist Scale, MoCA: Montreal Cognitive Assessment.

## Data Availability

Data are available upon request.

## Supplementary Materials

The following supporting information can be downloaded at: https://www.mdpi.com/article/10.3390/ijerph21020242/s1, Data S1: Administration, scoring and references for the tests and questionnaires used.
