# Supplementary material for: Safety and Efficacy of Pulmonary Rehabilitation for Long COVID Patients Experiencing Long-Lasting Symptoms
Source: Int J Environ Res Public Health. 2024 Feb 19;21(2):242. doi: 10.3390/ijerph21020242 (PMC10888408; doi:10.3390/ijerph21020242)
Supplement: Supplementary file 1 [file ijerph-21-00242-s001.zip › ijerph-2842997-supplementary.pdf]

Supplementary Data S1: Administration, scoring and references for the tests and questionnaires used.

Safety and Efficacy of Pulmonary Rehabilitation for Long COVID Patients Experiencing Long-Lasting Symptoms

Espérance Moine [1,*], Virginie Molinier [1], Adriana Castanyer [2], Amandine Calvat [3], Guillaume Coste [2], Antonin Vernet [2], Audrey Faugé [3], Perrine Magrina [3], Joan Lluis Aliaga-Parerra [2], Nicolas Oliver [1,3], François Alexandre [1] and Nelly Heraud [1]

[1] Direction de la Recherche clinique et de l'Innovation en Santé, Clariane, Lodève 34700, France
[2] Clinique du Souffle La Solane, Inicea, Osséja 66340, France
[3] Clinique du Souffle La Vallonie, Inicea, Lodève 34700, France
* Correspondence: esperance.moine@clariane.fr

Dyspnea

Dyspnea was measured with two different tools: the DYSPNEA-12 questionnaire [35,36] and the modified Medical Research Council dyspnoea scale (mMRC) [37].

The DYSPNEA-12 questionnaire includes 12 items and evaluates the physical and affective dimensions of dyspnea. Each item is measured using a score from 0 (none) to 3 (severe), and the total is calculated. The maximal score is 36, and the highest scores correspond to the highest levels of dyspnea severity.

The mMRC scale consists of five statements that describe almost the entire range of dyspnea from none (Grade 0) to almost complete incapacity (Grade 4).

Fatigue

The perceived fatigue was evaluated using the French version of the Multidimensional Fatigue Inventory (MFI-20) questionnaire [38,39]. It is a 20-item self-report questionnaire designed to measure fatigue and covers the following dimensions: General Fatigue, Physical Fatigue, Mental Fatigue, Reduced Motivation, and Reduced Activity. Each dimension comprises four questions; each of these questions is rated from 1 to 5. The highest scores correspond to the highest levels of fatigue. A subscore was calculated for each dimension by calculating the sum of the four questions, and a total fatigue score was calculated by calculating the sum of all the dimensions.

HADS

A validated French version of the Hospital Anxiety and Depression Scale (HADS) was used to assess psychological distress [40-42]. The HADS is a 14-item self-assessment scale for measuring distress with two subscales: anxiety and depression. The total HADS score ranges from 0 to 42, and the subscales from 0 to 21, with higher scores corresponding to higher levels of anxiety and depression.

PCLS

For post-traumatic stress disorder (PTSD) assessment, the Patient PTSD Checklist Scale (PCLS) was used [43] using the validated French version [44]. PCLS is a 17-item questionnaire. Each item is graded 1-5, and a higher score indicates a higher level of PTSD.

Nijmegen

The Nijmegen Questionnaire (NQ) was used to detect hyperventilation symptoms [45]. The NQ consists of 16 questions, with their answers scored from 0 (never) to 4 (very often).

MoCA

Cognitive impairment was assessed with the Montreal Cognitive Assessment (MoCA) test [46]. This questionnaire assesses attention, concentration, executive function skills, memory, language, visuocontructive skills, abstraction skills, calculation, and orientation. The execution time is approximately ten minutes, and the scale ranges from 0 to 30, with low scores indicating cognitive impairment.

Health-related quality of life

Health-related quality of life was measured with the SF-12 questionnaire [47]. The SF-12v2 is a health-related quality of life questionnaire consisting of twelve questions that measure eight health domains

to assess physical and mental health. Physical health-related domains include General Health, Physical Functioning, Role Physical, and Body Pain. Mental-health-related scales include Vitality, Social Functioning, Role Emotional, and Mental Health. A physical and a mental component score (PCS and MCS) can be calculated through the purchase of a license from QualityMetric. The PCS ranges from 11 to 60, and the MCS from 16 to 70, with higher values indicating higher health-related quality of life.

Exercise tolerance

Exercise tolerance was assessed with the 6-min walking test (6MWT). In accordance with good clinical practices and the guidelines of the American Thoracic Society [29], patients were instructed to walk as far as possible in 6 min in a 30 m corridor.

Muscle strength

Muscle strength was measured during maximal voluntary contraction of the quadriceps (QMVC) of the right leg [48]. The participants were seated on a dedicated ergometer for knee extensor testing (Quadriergoforme, Aleo Industrie, Salome, France) and equipped with a strain gauge torque sensor (Captels, Saint Mathieu de Treviers, France). The hip and the knee angle were set at 90°. The pelvis and the proximal extremity of the patella were securely attached to the chair in order to minimize movements of adjacent muscles. After a short warm-up consisting of repeating several submaximal contractions with visual feedback of the torque on a screen, patients had to perform three maximal voluntary contractions for 5s. The best of the three trials was noted as the maximal quadriceps torque. If the assessor observed a variation of more than 10% between the three trials, patients were asked to perform two additional maximal voluntary contractions.
